# Supplementary material for: Inequality in infrastructure access and its association with health disparities
Source: Nat Hum Behav. 2025 May 22;9(8):1669–82. doi: 10.1038/s41562-025-02208-3 (PMC12367552; doi:10.1038/s41562-025-02208-3)
Supplement: Supplementary file 1 — Supplementary Information sections 1–5, Figs. 1–15, Tables 1–7 and References. [file 41562_2025_2208_MOESM1_ESM.pdf]

nature human behaviour

Supplementary information https://doi.org/10.1038/s41562-025-02208-3

Inequality in infrastructure access and its association with health disparities

In the format provided by the authors and unedited

Table of Contents

Supplementary Section 1. Definition and classification of infrastructure 2
Supplementary Section 2. Illustration of the human-infrastructure interaction......................... 5
Supplementary Section 3. Data acquisition and processing 6
Supplementary Section 4. Calculation of infrastructure access inequalities 10
Supplementary Section 5. Sensitivity analyses.. 13
Supplementary Figures 1-15 16
Supplementary Tables 1-7 31
Supplementary references 38

1. Definition and classification of infrastructure

Infrastructure is essential for supporting economic activities, social well-being, and human development. According to the Cambridge Dictionary, infrastructure refers to "the basic systems and services, such as transport and power supplies, that a country or organization uses in order to work effectively". As early as the 18th century, economists began documenting the socioeconomic nature of infrastructure. Adam Smith, for example, justified the principle of the "invisible hand of the market" while assigning the state the role of infrastructure investor, emphasizing the state's obligation to maintain public facilities and institutions[1]. The term "infrastructure" was first used by the military to describe war logistics during the Second World War and has since expanded to various fields[2]. In his famous book of *The Strategy of Economic Development*, German economist Albert Hirschman defined infrastructure as "capital that provides public services", highlighting two key elements: capitalness and publicness[3]. Buhr[4] further described infrastructure as "the sum of all relevant economic data such as rules, stocks, and measures with the function of mobilizing the economic potentialities of economic agents". More recently, Fulmer[5] defined infrastructure as "the physical components of interrelated systems providing commodities and services essential to enable, sustain, or enhance societal living conditions". Despite its varying definitions, infrastructure is widely recognized as both the fundamental physical and organizational systems necessary for the functioning of society and the economy[6, 7, 8]. In this article, we focus on the physical components of infrastructure, as they are more tangible and straightforward to measure.

As reviewed in previous studies, infrastructure can encompass a wide range of types[6, 7, 8]. Generally, two main criteria are used for classification: features and functions. Under the prior criterion, research often focuses on the structure or material nature of infrastructure. For instance, Biehl[9] classified infrastructure into network (e.g., roads, railways, electrical

facilities) and point (e.g., schools, hospitals, museums), depending on whether active human involvement is required for the operation of a structure. Similarly, infrastructure was classified infrastructure into tangible (hard) and intangible (soft) assets based on their physical nature[10].

Another approach classifies infrastructure according to its functional role. Hansen[11] distinguished between economic and social infrastructure based on its direct or indirect influence on regional economic development, a classification widely used in the literature[12, 13, 14, 15]. Researchers have also differentiated between "core" and "non-core" infrastructure based on their importance to the economy's sustainable functioning. Sturm and Jacobs[16] used a similar distinction between "basic" and "complementary" infrastructure. Jochimsen[17] categorized infrastructure into three kinds: personal (which shapes the values of economic agents), institutional (which promotes social integration), and material (which addresses physical and social needs).

Moreover, some studies have adopted a dual classification approach, considering both features and functions. Vaughan-Morris[18] described infrastructure as "hard" and "soft" types, where hard infrastructure includes economic, social, and industrial facilities, while soft infrastructure refers primarily to institutions and intangible assets, such as government buildings, laws, regulations. Despite variations in classification systems, the majority incorporate critical infrastructure systems that are essential to societal operation, such as transportation, communications, energy supply, healthcare, and sanitation systems[6, 7, 8].

In recent years, there has been a growing recognition of the role that the natural environment, such as green and water infrastructure[19, 20, 21, 22], plays in human and social development. Frischmann[23] argued that the natural environment functions similarly to traditional infrastructure, serving as a vital input for a wide range of human and natural goods and

services. He introduced the concept of "environmental infrastructure", highlighting its essential role in supporting various processes and services. Consequently, recent studies and reports have called for a broader integration of environmental considerations into infrastructure design and management to maintain human health and social well-being. For instance, the World Bank's report *"Putting Nature to Work: Integrating Green and Gray Infrastructure for Water Security and Climate Resilience"* discussed the role of nature-based solutions in boosting urban resilience, reducing pollution, and mitigating climate change[24]. Likewise, the Organisation for Economic Co-operation and Development (OECD), in its report *"Financing Climate Futures: Rethinking Infrastructure"*, stressed the importance of investing in green infrastructure and integrating climate considerations into infrastructure planning for long-term sustainability[25].

2. Illustration of the human-infrastructure interaction

We selected Australia and Burkina Faso, two representative countries of the Global North and Global South, respectively, as examples to illustrate how populations interact with their surrounding infrastructure and how this could affect infrastructure access measure in our population-weighted exposure model. Even though these two countries have similar population sizes, they show significant differences in their provision of infrastructure to human beings (Supplementary Fig. 14). The average infrastructure values, computed from the generated global infrastructure maps, were 0.29, 0.00, 0.01 for Australia and 0.59, 0.01, 0.00 for Burkina Faso for economic, social, and environmental dimensions, respectively. After applying the population weights, the disparities in infrastructure access were amplified (Supplementary Fig. 14). Australia demonstrated a significant increase in infrastructure access across all three dimensions compared to the raw infrastructure values, whereas Burkina Faso showed a decrease from 0.59 to 0.20 in economic infrastructure access, along with slight increases in other two dimensions. Spatially, the distribution of the Australian population closely matched that of the infrastructure, with most of the population concentrated in southeast coastal areas, where infrastructure was also predominantly located (Supplementary Fig. 14). Consequently, the comprehensive infrastructure access measure in Australia exceeds the original infrastructure values.

3. Data acquisition and processing

Critical infrastructure data

We used the global harmonized dataset of critical infrastructure (CI) as a baseline map to derive our economic-social-environmental infrastructure maps. The CI dataset aggregated high-resolution geospatial OpenStreetMap (OSM) data of 39 infrastructure types that are categorized under seven overarching CI systems (Supplementary Table 4) to consistent raster layers at 0.1° × 0.1° and 0.25° × 0.25° spatial resolutions[26]. We acquired data on the amount of infrastructure for each CI type in 2020 with a resolution of 0.1° × 0.1°.

Greenspace

The European Space Agency's 10-m global land cover product (WorldCover) for 2020 was introduced to quantify the spatial distribution of greenspace. Produced by the joint use of Sentinel-1 and Sentinel-2 satellite imagery, the WorldCover map provides reliable land cover information under 11 specific classes with an overall accuracy of 75%[27]. Following Chen, Wu[28], we extracted all types of forest, shrub, grass, herbaceous wetland, and mangrove from the WorldCover map as greenspaces.

Air pollution

The NASA's Goddard Earth Observing System Composition Forecast (GEOS-CF) data[29] was adopted to quantify the spatial distribution of fine particulate matter ($PM_{2.5}$). Using the Google Earth Engine (GEE) cloud computing platform[30], we aggregated all the GEOS-CF hourly observations acquired in 2020 and calculated the annual mean $PM_{2.5}$ values based on the "PM25_RH35_GCC" band. After that, we normalized the data to 0-1 by dividing the maximum $PM_{2.5}$ value.

Heat duration

The ERA5 climate reanalysis data[31] were used to quantify the spatial distribution of heat duration. Specifically, we calculated the 90% percentile value of the 2-m air temperature band ("temperature_2m") of daily ERA5 aggregates between June-August 2020 on GEE. We then applied the value (27.73°) as a threshold to all daily data in 2020 and calculated the number of heat days for each 0.1° × 0.1° pixel (which was recognized as yes if the 2-m air temperature was greater than the threshold). Finally, we normalized the value of heat duration layer to 0-1.

Nighttime lights

Nighttime light data collected by the NASA/NOAA Visible Infrared Imaging Radiometer Suite (VIIRS) was used as a proxy of socioeconomic activities. This data offers global daily measurements of nocturnal visible and near-infrared light at 15 arcseconds (~500m at the Equator)[32, 33]. To remove extraneous features such as biomass burning and stray light, we used the annual global VIIRS nighttime lights (VNL) V2 product that had been processed on monthly cloud-free average radiances through outlier removal, aurora filter, and annual mosaicking[34]. We downloaded the annual average VNL for 2020 from the Earth Observation Group and further aggregated it into 0.1° × 0.1° using the bilinear interpolation tool in ArcMap.

Global urban areas

We use the global urban boundary (GUB) data to delineate the urban extent of cities. This dataset was extracted from 30-m Landsat imagery using a hierarchical approach to improve the homogeneity of built-up areas in urban centres and to maintain the heterogeneity of built-up areas at the urban fringes[35]. Different from commonly used administrative boundaries, GUBs represent a physical region that consists of not only built-up areas but also associated natural lands in urban centres such as greenspace and water bodies[36]. To ensure a sufficient

sample size for subsequent analyses, we selected GUBs with a geographic area greater than 100 km^2, resulting in a total of 1028 urban areas globally.

Global administrative unit layers

The Global Administrative Unit Layers (GAULs) from the Food and Agriculture Organization (FAO) of the United Nations were used as hierarchy units for the spatial analysis of global infrastructure access at country and county levels. GAULs represent the compilation and dissemination of the best available information on administrative units for all countries in the world, providing a contribution to the standardization of the spatial dataset representing administrative units[37].

Global North and Global South countries

The concept of Global North and Global South is used to describe a grouping of countries under different socioeconomic and political characteristics. Generally, the Global North correlates with the Western world which tends to be wealthier and less unequal, while the Global South corresponds to developing countries that share a set of vulnerabilities and challenges[38]. In this study, the names of countries in the Global South were obtained from the Organization for Women in Science for the Developing World (OWSD, https://owsd.net/). In total, we examined 166 countries with 54 in the Global North and 112 in the Global South.

Socioeconomic data

Data on gross domestic product (GDP) for each country in 2020 were collected from the World Bank (https://data.worldbank.org). Data on Human Development Index (HDI) for each country in 2020 were retrieved from United Nations Development Programme (https://hdr.undp.org/).

Population

We used the WorldPop dataset for 2020 to quantify the spatially explicit distribution of population. WorldPop provides the estimated number of people residing in each 100 × 100 m grid based on a random forest model and a global database of administrative unit-based census information[39], which has much finer spatial resolution and update frequency than other population datasets such as the GWP[40] and LandScan[41]. We aggregated WorldPop data to the 0.1° × 0.1° spatial resolution that aligns with the infrastructure data.

Health data

Health data on health-adjusted life expectancy (HALE) and disability-adjusted life years (DALYs) for each country in 2020 were accessed from Institute for Health Metrics and Evaluation (IHME) at the University of Washington (https://vizhub.healthdata.org/gbd-results/). According to the World Health Organization (WHO), HALE is defined as the average number of years that a person can expect to live in "full health" by taking into account years lived in less than full health due to disease and/or injury[42]. As for DALY, it is a measure of overall disease burden, expressed as the number of years lost due to ill-health, disability, or early death[43]. DALYs are calculated as the sum of the years of life lost to due to premature mortality (YLLs) and the years lived with a disability (YLDs) due to prevalent cases of the disease or health condition in a population.

4. Calculation of infrastructure access inequalities

In this study, we introduced two pivotal indices namely the Gini coefficient (*Gini*) and the Inequality index (*Inq*) for assessing the extent of inequality in human access to infrastructure on a national level. Served as quantitative summaries, these metrics capture the statistical distribution of infrastructure access and thereby provide insights into the overall inequality landscape.

Gini coefficient

The Gini coefficient is a relative measure of the difference between the current distribution and the state of everyone receiving the exact same value ("perfect" equality)[44]. It is derived from the Lorenz curve, which plots the cumulative infrastructure access of a population from the lowest to the highest infrastructure access, and compares it to a perfectly equal distribution of infrastructure access[45]. Mathematically, the Gini coefficient quantifies the extent of inequality by calculating the area between the Lorenz curve and the line representing perfect equality (Supplementary Fig. 15). This calculation involves determining the ratio of the area bounded by the line of equality and the Lorenz curve (region *A*) to the total area beneath the line of equality (region *A* plus region *B*):

$$Gini = \frac{Area_A}{Area_A + Area_B} \tag{S1}$$

where $Area_A$ and $Area_B$ represent the areas of regions *A* and *B*, respectively. Given that the scales of both the x- and y-axes in Supplementary Fig. 15 span from 0 to 1, it follows that $Area_A + Area_B = 0.5$. Consequently, the Gini coefficient can be formulated as:

$$Gini = \frac{Area_A}{0.5} = \frac{0.5 - Area_B}{0.5} = 1 - 2 \times Area_B \tag{S2}$$

Our focus now shifts to the computation of the area pertaining to region B, which is delimited by the cumulative proportion of infrastructure access and the cumulative proportion of residents, spanning from those with the least infrastructure access to those with the highest. To achieve this, we initially employed a method of numerical integration to determine the area of each trapezoidal segment B_i contributed by i-th residents. The area of trapezoid B_i ($Area_{Bi}$) is calculated as follows:

$$Area_{B_i} = \frac{1}{2} \times \left(\frac{\sum_{j=1}^{i-1} I_j}{\sum_{j=1}^{n} I_j} + \frac{\sum_{j=1}^{i} I_j}{\sum_{j=1}^{n} I_j} \right) \times \frac{1}{n} \tag{S3}$$

where I_j is the infrastructure that is exposed to j-th resident and n is the total resident number.

Subsequently, we aggregated the area of each trapezoidal segment across all residents, where the area of region B is calculated as:

$$Area_B = \sum_{i=1}^{n} Area_{B_i} = \sum_{i=1}^{n} \frac{1}{2} \times \left(\frac{\sum_{j=1}^{i-1} I_j}{\sum_{j=1}^{n} I_j} + \frac{\sum_{j=1}^{i} I_j}{\sum_{j=1}^{n} I_j} \right) \times \frac{1}{n} \tag{S4}$$

By substituting Eq. (S4) into Eq. (S2), we finally derive the Gini coefficient using the following formula.

$$Gini = 1 - \frac{\sum_{i=1}^{n} \sum_{j=1}^{i-1} I_j + \sum_{i=1}^{n} \sum_{j=1}^{i} I_j}{n \times \sum_{j=1}^{n} I_j} \tag{S5}$$

A higher Gini coefficient indicates greater inequality, with 0 representing perfect equality and 1 representing perfect inequality[46].

Inequality index

The Inequality index (*Inq*) is another complementary measure of the spatial inequality in infrastructure access[47, 48]. It examines how between-region heterogeneities vary with the overall mean of infrastructure distributions[49].

$$Inq = \frac{\sigma}{\sqrt{\mu(1-\mu)}};\ 0 < \mu < 1 \tag{S6}$$

where μ and σ signify mean and standard deviation values of county-scale infrastructure access within each country. Analogous to the Gini coefficient, *Inq* also ranges from 0 to 1, with 0 representing absolute equality and 1 representing absolute inequality.

5. Sensitivity analyses

Adaptability of the economic-social-environmental infrastructure framework

Our economic-social-environmental infrastructure framework is designed to be flexible, which can accommodate a wide range of critical infrastructure systems based on different research and application needs. To illustrate this adaptability, we have incorporated water and waste infrastructure into our economic and social infrastructure categories and compared the country-level access values with previous versions (Supplementary Fig. 13). In these plots, each point represents a single country, with the x-axis showing the combined economic/social, water, and waste infrastructure access values, and the y-axis representing economic/social infrastructure access alone.

The results reveal a high correlation between them, with R^2 values of 0.97 for economic infrastructure and 0.95 for social infrastructure. This indicates that the inclusion of water and waste infrastructure has a limited impact on the overall socioeconomic access metrics, probably due to the relatively low density of these systems compared to other critical infrastructure systems (Supplementary Fig. 8). Statistically, most pixels (> 95%) in the water and waste infrastructure data have a value of 0 (Supplementary Table 5).

Interdependencies between infrastructure access and its association with human health

The interdependences between different types of infrastructure may play an important role in shaping access rates, realistic benefit, and associated health outcomes. To explore this, we first calculated Pearson correlation coefficients for human access to each of the seven critical infrastructure (CI) systems at the country level (Supplementary Fig. 7). The average correlation across these systems is 0.45, with transportation-health, energy-transportation, and transportation-education access showing the highest correlations at 0.67, 0.64, and 0.55, respectively. In contrast, water and waste infrastructures show weaker correlations with other

CI systems. This suggests that while certain infrastructure systems, such as transportation and energy, are closely interlinked and may mutually reinforce access and benefits, water and waste infrastructures operate more independently.

Next, we examined the association between infrastructure interdependencies and health-adjusted life expectancy (HALE), focusing on energy, transportation, and health infrastructure given their relatively high correlations. In addition to examining individual access variables, we included interaction terms for each pair of infrastructure access variables in our regression models. For example, the term *Energy*Transportation* represents combined access to energy and transportation infrastructure (Eqs. (S7-10)). The coefficients of these interaction terms indicate whether the combined impact of the two infrastructures on health outcomes is stronger (>0) or weaker (<0) than the sum of their individual effects. In addition, we included covariate variables of the logarithmic value of population (*LnPop*), the logarithmic value of population (*LnGDP*) for country *j* in group *g* (i.e., Global North or Global South).

$$HALE_{j,g} = \beta_0 + \gamma_g + \beta_1 LnPop_{j,g} + \beta_2 LnGDP_{j,g} + \beta_3 Energy_{j,g} + \beta_4 Transportation_{j,g} + \beta_5 Energy_{j,g} * Transportation_{j,g} + \varepsilon_{i,g} \quad \text{(S7)}$$

$$HALE_{j,g} = \beta_0 + \gamma_g + \beta_1 LnPop_{j,g} + \beta_2 LnGDP_{j,g} + \beta_3 Energy_{j,g} + \beta_4 Health_{j,g} + \beta_5 Energy_{j,g} * Health_{j,g} + \varepsilon_{i,g} \quad \text{(S8)}$$

$$HALE_{j,g} = \beta_0 + \gamma_g + \beta_1 LnPop_{j,g} + \beta_2 LnGDP_{j,g} + \beta_3 Transportation_{j,g} + \beta_4 Health_{j,g} + \beta_5 Transportation_{j,g} * Health_{j,g} + \varepsilon_{i,g} \quad \text{(S9)}$$

$$HALE_{j,g} = \beta_0 + \gamma_g + \beta_1 LnPop_{j,g} + \beta_2 LnGDP_{j,g} + \beta_3 Energy_{j,g} + \beta_4 Transportation_{j,g} + \beta_5 Health_{j,g} + \beta_6 Energy_{j,g} * Transportation_{j,g} + \beta_7 Energy_{j,g} * Health_{j,g} + \beta_8 Transportation_{j,g} * Health_{j,g} + \varepsilon_{i,g} \quad \text{(S10)}$$

Our results show that greater access to energy, transportation, and health infrastructure alone is linked to improved health outcomes (Supplementary Table 6). Regarding their combined effects, we found that the interaction between energy and transportation infrastructure access is negatively associated with health, suggesting a weaker influence compared to their individual effects. Conversely, the joint impact of transportation and health infrastructure is significantly associated with increased HALE, indicating that people with greater access to both tend to have longer life expectancy. However, no significant correlation was found between the interaction of energy and health infrastructure access and HALE.

Supplementary figures

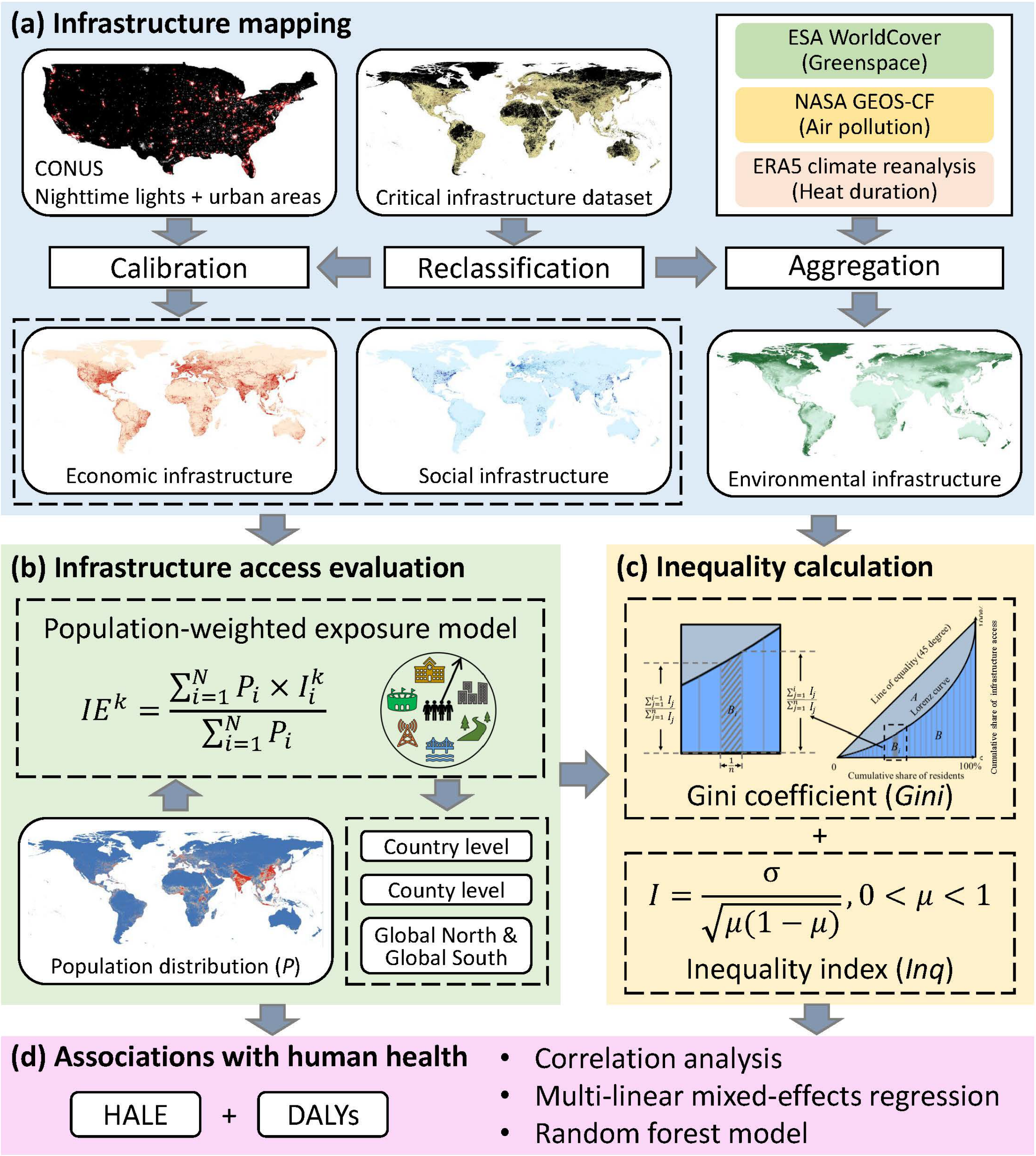

Supplementary Fig. 1. Flowchart of the research design with four major steps. (a) Global mapping of economic, social, and environmental infrastructure. (b) Assessing differences in human access to infrastructure at the country and county levels and in Global North and South countries. (c) Measuring inequality in infrastructure access using the Gini coefficient and Inequality index. (d) Exploring the impacts of infrastructure access and inequality on human health.

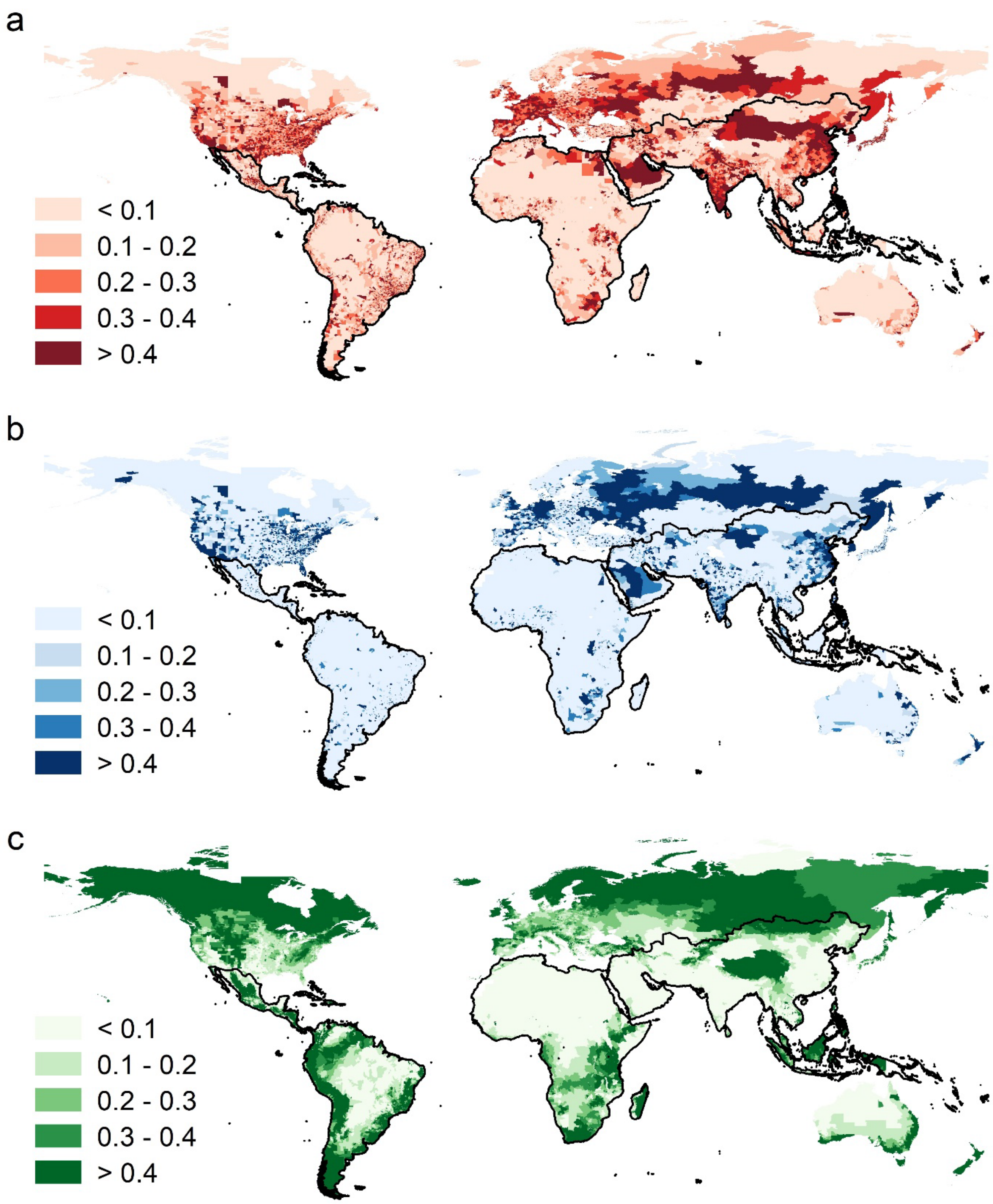

Supplementary Fig. 2. County-level human access to (a) economic, (b) social, and (c) environmental infrastructure distributions across the globe. Black boundaries indicate countries in the Global South.

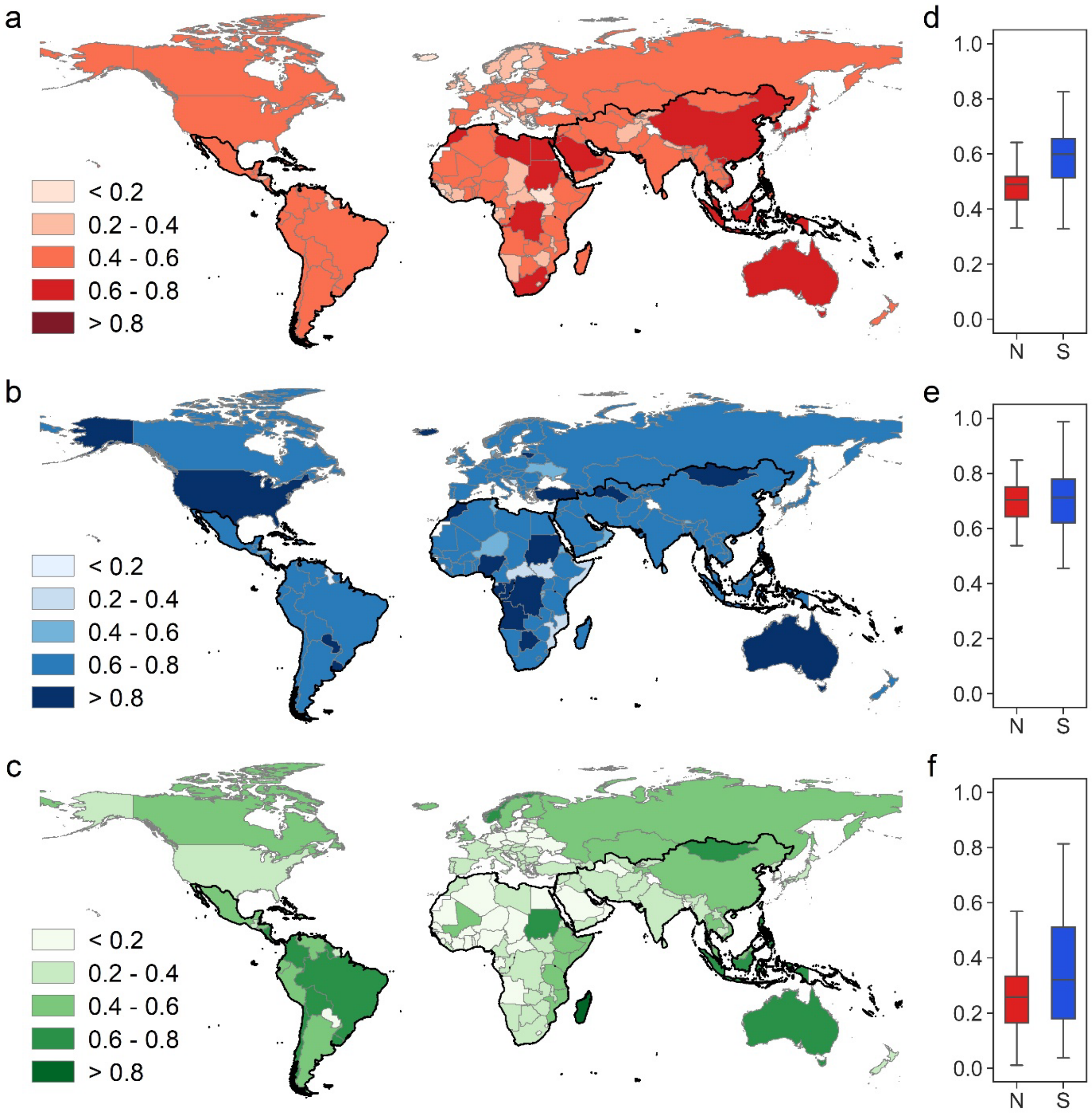

Supplementary Fig. 3. Country-level infrastructure access inequalities measured by the inequality index across the globe. (a-c) Global maps of (a) economic, (b) social, and (c) environmental infrastructure access inequalities. Black boundaries indicate countries in the Global South. (d-f) Box plots of (d) economic, (e) social, and (f) environmental infrastructure access inequalities in countries in the Global North (N) and Global South (S). Box plots display the distribution of data, with the median (50th percentile) at the center, the interquartile range (25th to 75th percentiles) as the box, and whiskers extending to the maximum and minimum values within 1.5 times the interquartile range. Sample sizes: Global North = 53 countries, Global South = 111 countries. No replicates were carried out and no adjustments for multiple comparisons were applied. A two-sided t-test was used to compare infrastructure access inequalities between the Global North and Global South: economic: $t(162)=-1.99$, $p=0.048$, Cohen's $d=-0.35$, 95% CI=[-0.08, -0.00]; social: $t(162)=0.68$, $p=0.500$, Cohen's $d=0.12$, 95% CI=[-0.03, 0.06]; environmental: $t(162)=-2.20$, $p=0.029$, Cohen's $d=-0.39$, 95% CI=[-0.13, -0.02]).

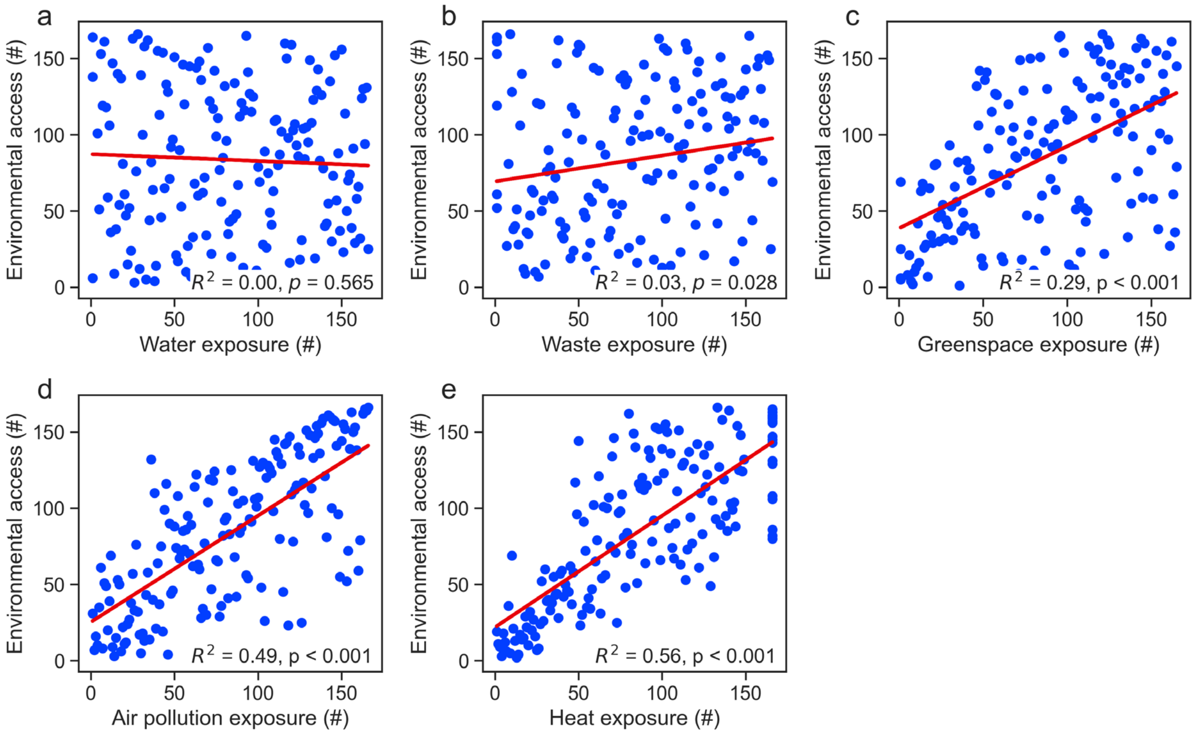

Supplementary Fig. 4. Scatter plots comparing the rank of overall environmental infrastructure access with the rank of single environmental factor exposures at the country level: (a) water, (b) waste, (c) greenspace, (d) air pollution, and (e) heat. Ordinary least squares (OLS) linear regression models were conducted with two-sided hypothesis tests and no multiple comparison adjustments. Blue points represent individual data values, while red lines indicate the predicted values from the OLS models. The coefficient of determination (R^2) and p value are reported for each model. The sample size for each plot is n=166.

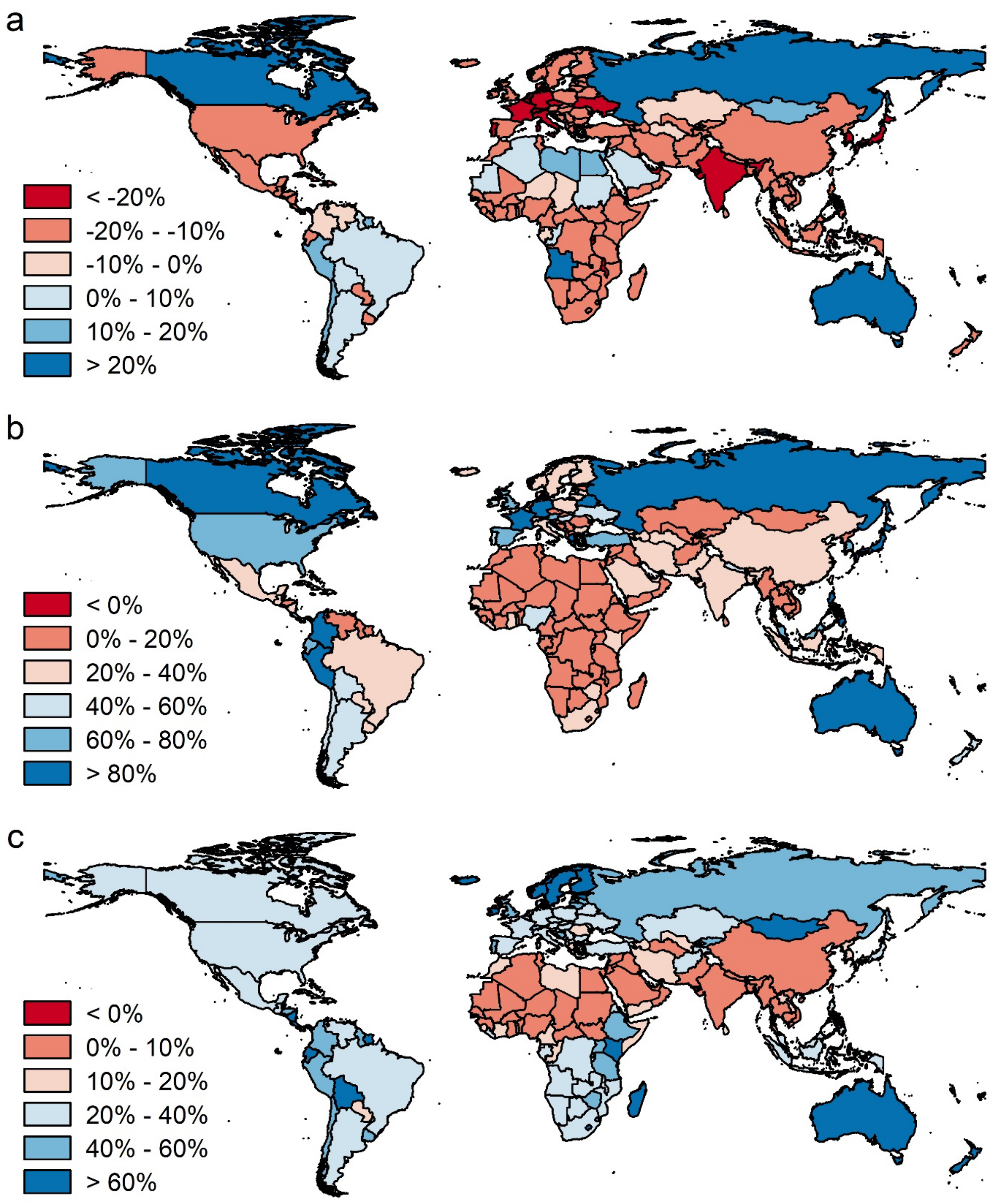

Supplementary Fig. 5. Country-level differences between population-weighted infrastructure access and infrastructure values for (a) economic, (b) social, and (c) environmental dimensions.

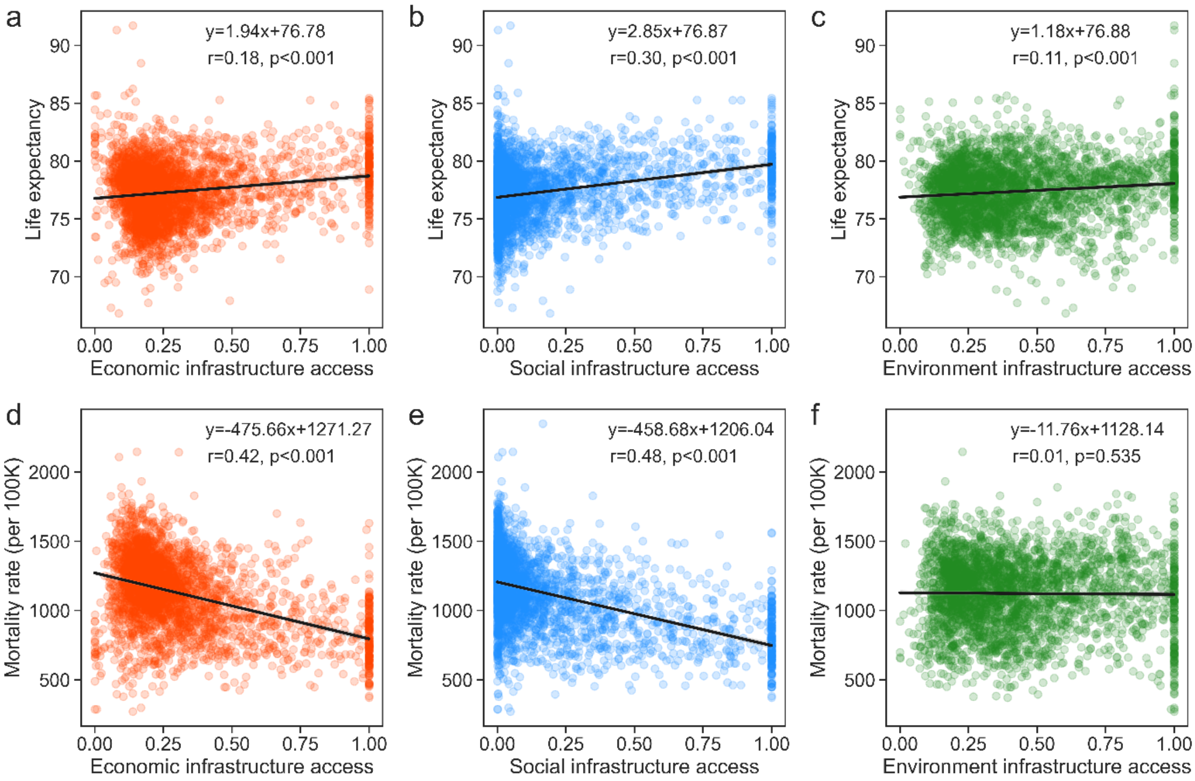

Supplementary Fig. 6. Scatter plots between life expectancy and infrastructure access (a-c) and mortality rate (per 100,000 population) and infrastructure access (d-f) for counties in the United States. All axes are labelled, and colour scales distinguish different types of infrastructure. Panels from left to right denote economic, social, and environmental infrastructure, respectively. Ordinary least squares (OLS) linear regression models were conducted with two-sided hypothesis tests and no multiple comparison adjustments. Points represent individual data values, while black lines indicate the predicted values from the OLS models. The regression equation, correlation coefficient (r), and p value are reported for each model. The sample size for each plot is n=3104.

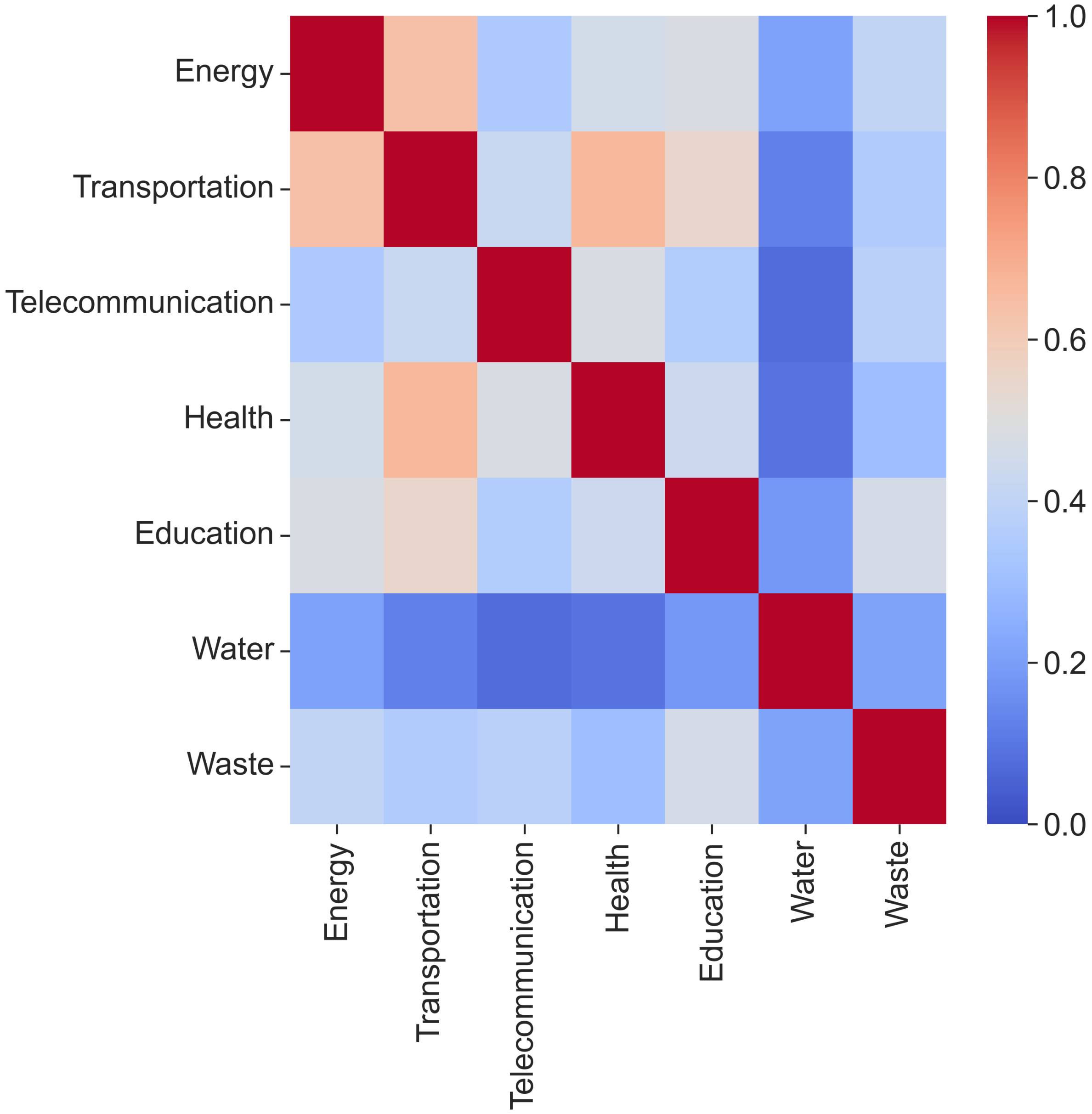

Supplementary Fig. 7. Heatmap showing Pearson correlation coefficients for access to each of the seven critical infrastructure systems at the country level. Pearson correlation coefficients were computed for each pairwise comparison, and corresponding p-values were calculated using two-sided hypothesis tests. Exact p-values are provided in Supplementary Table 7. The sample size for each correlation analysis is n=166.

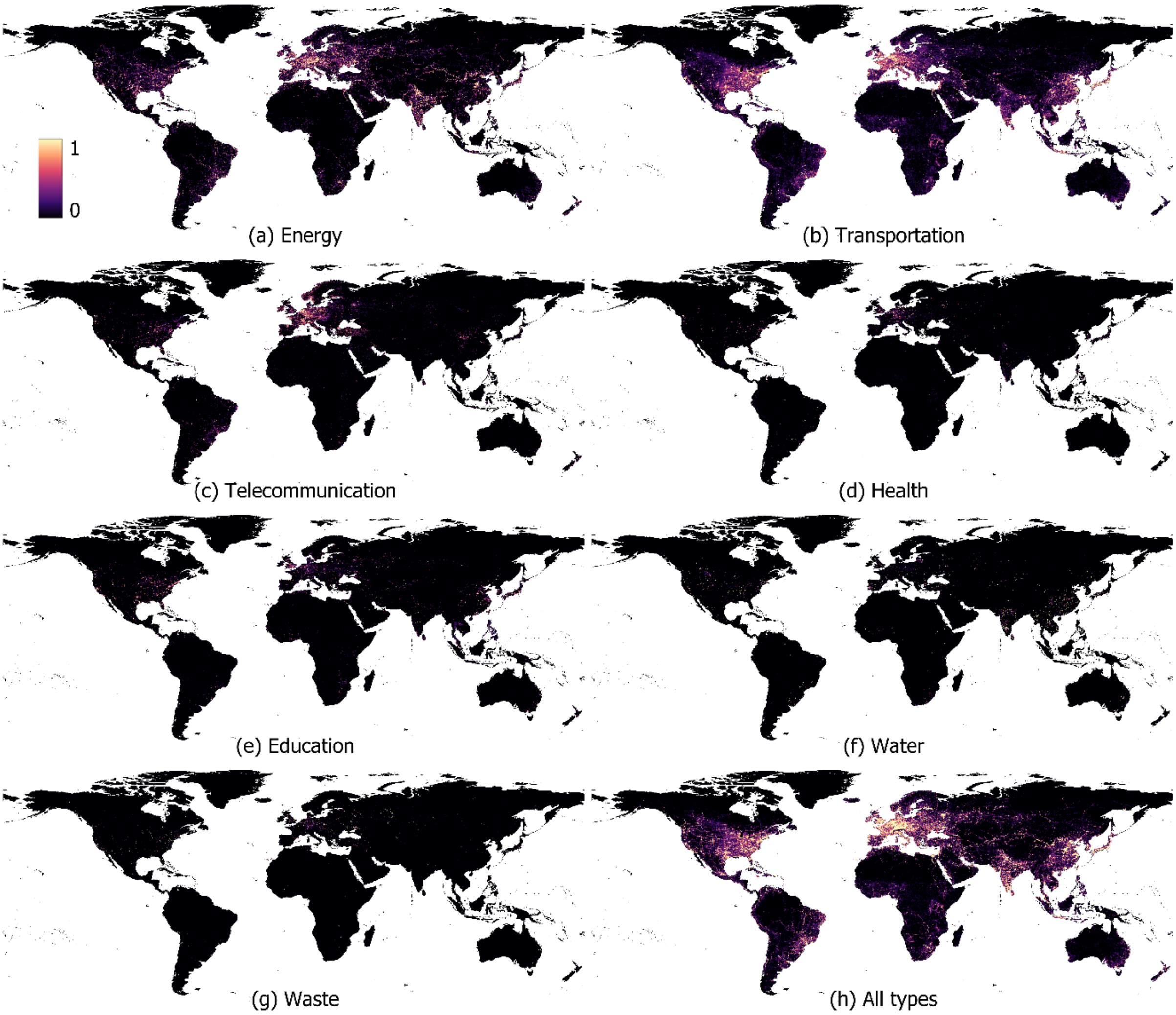

Supplementary Fig. 8. Maps of critical infrastructure distribution for energy, transportation, telecommunication, health, education, water, and waste systems (a-g). For each system, the number of infrastructure types was normalized to a range of 0-1 and then averaged (see Supplementary Table 5 for the correspondence between infrastructure systems and types). Map (h) shows the normalized average across all 39 infrastructure types.

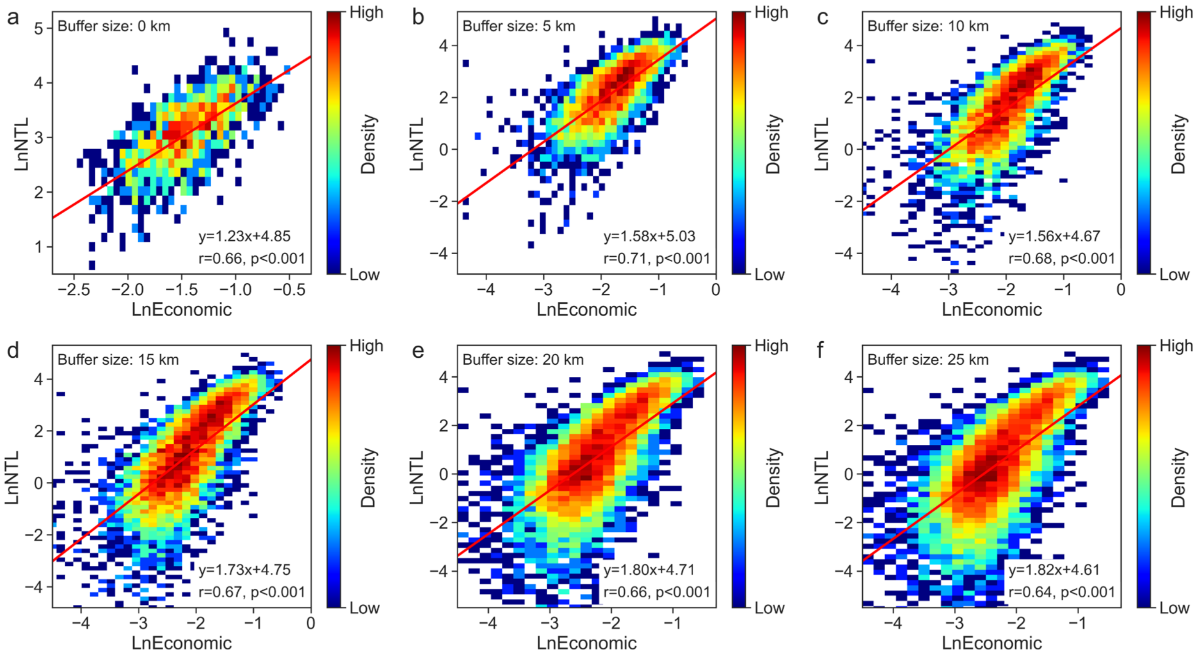

Supplementary Fig. 9. Correlations between logarithmic values of nighttime lights (LnNTL) and logarithmic values of economic infrastructure (LnEconomic) in the continental United States at a 0.1° × 0.1° spatial resolution, with buffer sizes around urban areas ranging from 5 km to 25 km. Ordinary least squares (OLS) linear regression models were conducted with two-sided hypothesis tests and no adjustments for multiple comparisons. The density of data points is represented using a colour gradient, while red lines indicate the predicted values from the OLS models. The regression equation, correlation coefficient (r), and p-value are reported for each model. Sample sizes for each plot: (a) n = 4,192, (b) n = 12,612, (c) n = 19,384, (d) n = 26,308, (e) n = 33,308, (f) n = 40,728.

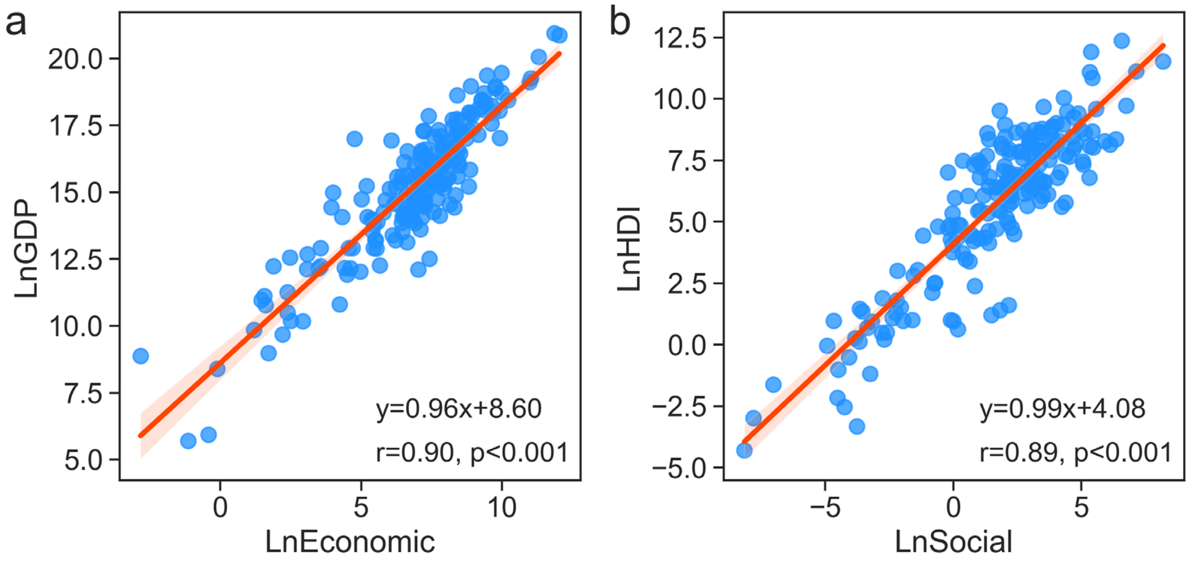

Supplementary Fig. 10. Validation of economic and social infrastructure at the country level. (a) Scatter plots between logarithmic values of GDP (LnGDP) and logarithmic values of economic infrastructure (LnEconomic). (b) Scatter plots between logarithmic values of HDI (LnHDI) and logarithmic values of social infrastructure (LnSocial). Individual data points are shown as overlaid dot plots. Ordinary least squares (OLS) linear regression models were conducted with two-sided hypothesis tests, and no adjustments were made for multiple comparisons. Shadows denote the 95% confidence interval level of linear regression models, with the center of the error bands indicating the predicted values. The regression equation, correlation coefficient (r), and p-value are reported for each model. Sample sizes for each plot: (a) n=185 and (b) n=196.

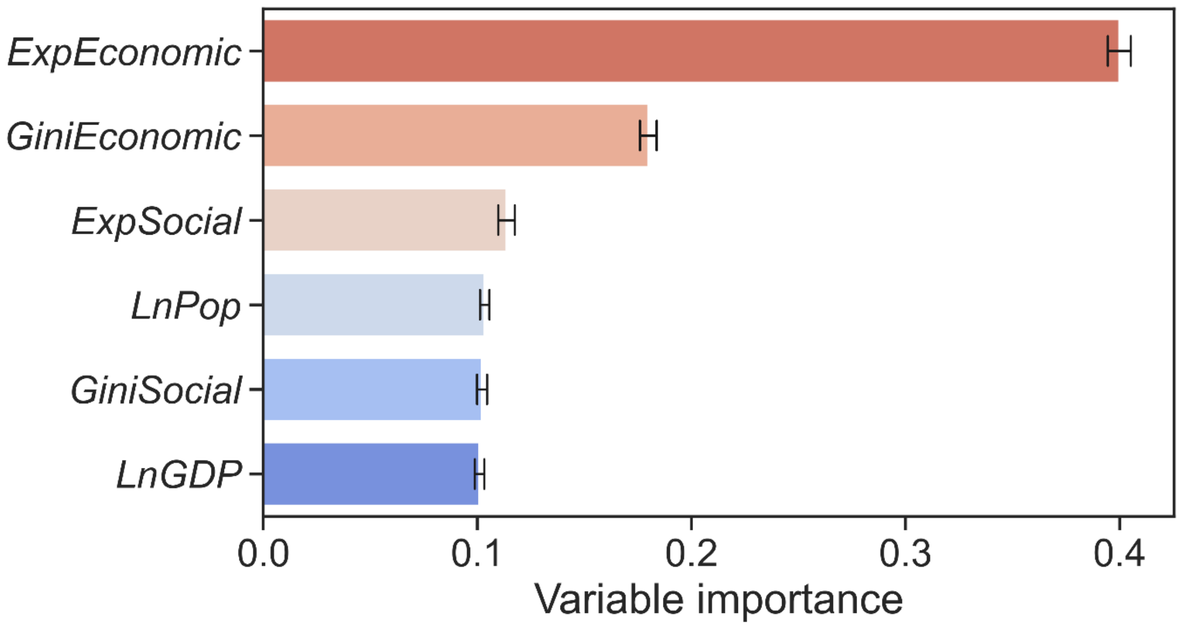

Supplementary Fig. 11. Variable importance of random forest models taking health-adjusted life expectancy (HALE) as the response variable. The models were iteratively executed 100 times and error bars indicated the standard deviations.

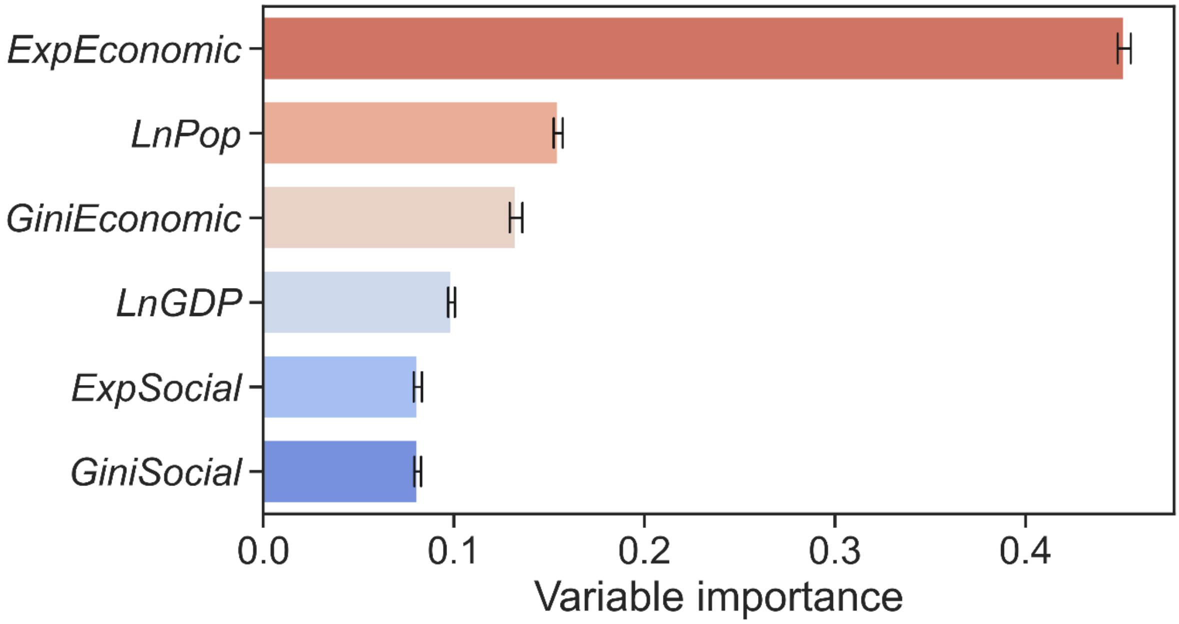

Supplementary Fig. 12. Variable importance of random forest models taking logarithmic values of disability-adjusted life years (DALYs) as the response variable. The models were iteratively executed 100 times and error bars indicated the standard deviations.

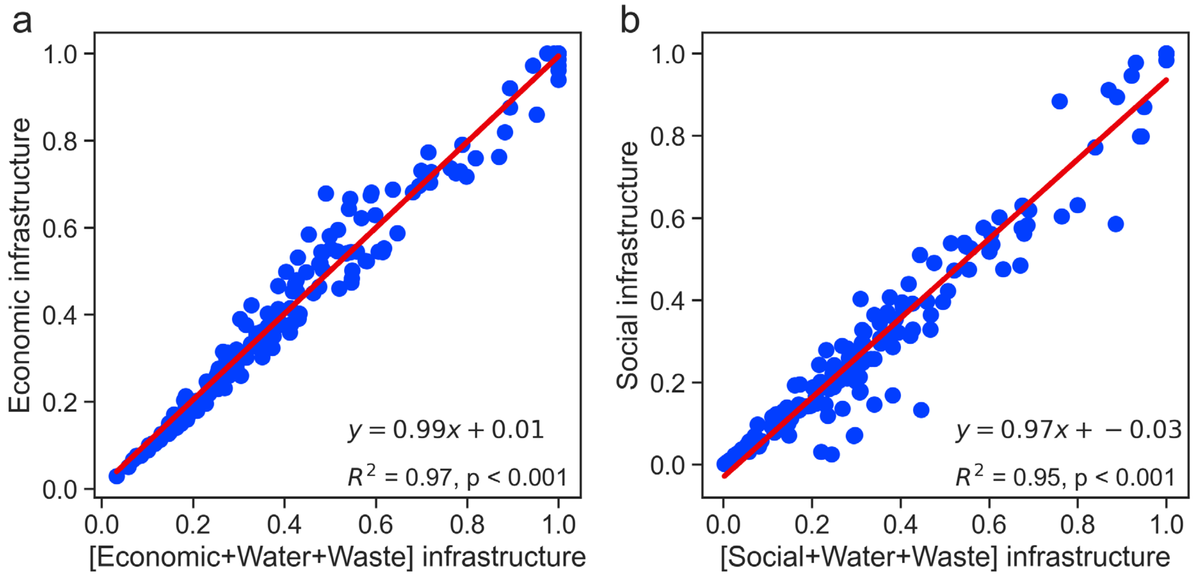

Supplementary Fig. 13. Scatter plots comparing the access values of economic/social, water, and waste infrastructure with economic/social infrastructure alone at the country level. Ordinary least squares (OLS) linear regression models were conducted with two-sided hypothesis tests and no multiple comparison adjustments. Blue points represent individual data values, while red lines indicate the predicted values from the OLS models. The coefficient of determination (R^2) and p value are reported for each model. The sample size for each plot is n=166.

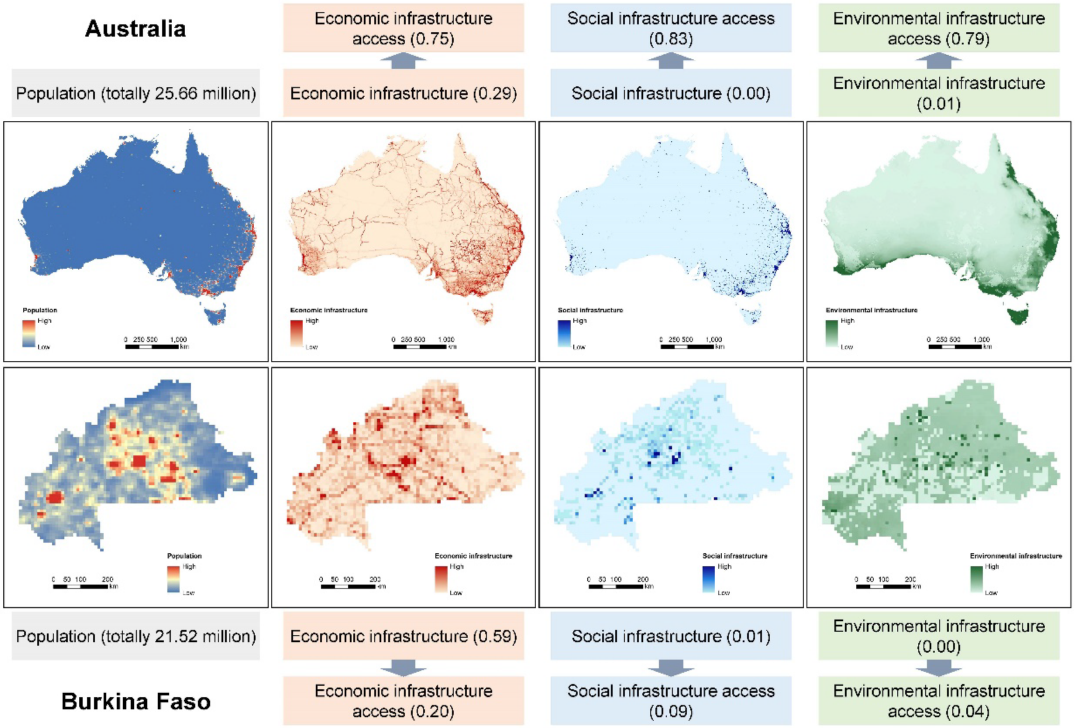

Supplementary Fig. 14. Comparison of population distribution, infrastructure distribution, and infrastructure access in Australia (upper) and Burkina Faso (lower). Panels from left to right represent 0.1° × 0.1° spatial maps of population, economic infrastructure, social infrastructure, and environmental infrastructure, respectively. Values in the parentheses represent the average infrastructure or infrastructure access values at the country level.

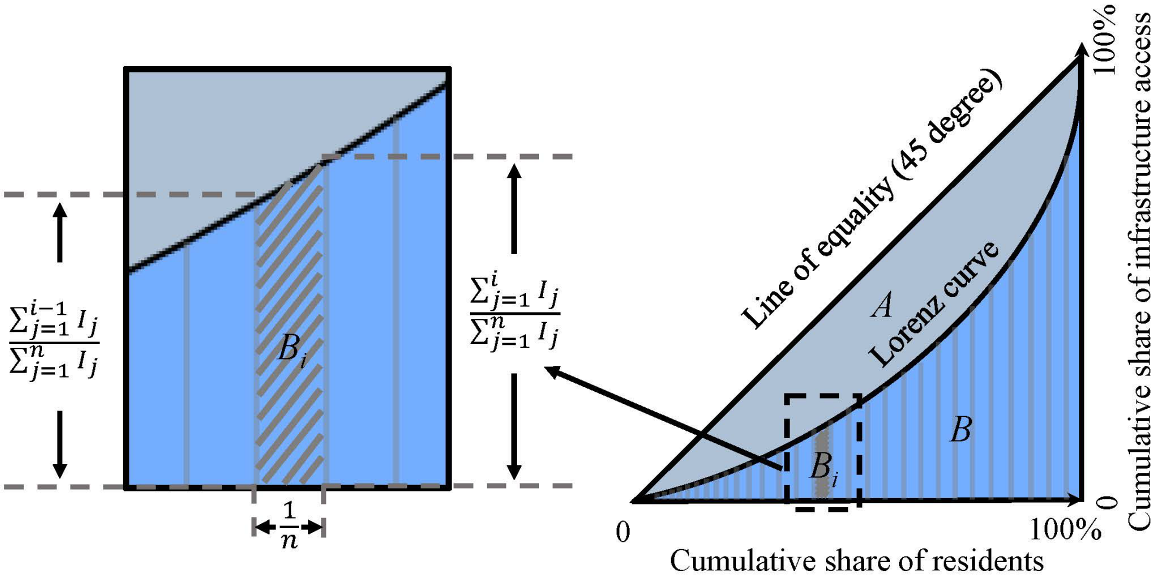

Supplementary Fig. 15. Illustrative diagram of Gini coefficient-based inequality assessments of infrastructure access. y-axis shows the cumulative share of infrastructure access and x-axis shows the cumulative share of residents from the lowest to the highest infrastructure access. The Gini coefficient is defined as the ratio of the area that lies between the line of equality and the Lorenz curve (region A) over the total area under the line of equality (region A plus region B), where the Lorenz curve plots the proportion of the infrastructure access (y-axis) that is cumulatively shared by the residents (x-axis). B_i indicates the contribution of i-th residents to the accumulated infrastructure access and is estimated by the trapezoid area as shown in the left panel, where I_j represents the infrastructure that is exposed to the j-th resident and n represents the total resident number.

Supplementary tables

Supplementary Table 1. Statistics of county-level human access to economic, social, and environmental infrastructures across regions. Values are presented as mean ± standard deviation. We conducted two-sided one-way ANOVA tests to examine differences in infrastructure access across the three dimensions (economic, social, and environmental) and regions (Europe, Asia, North America, South America, Oceania, Africa), and a two-sided t-test to compare infrastructure access between the Global North and Global South.

Region	Count	Infrastructure access		
		Economic	Social	Environmental
Global North	10780	0.31±0.27	0.21±0.32	0.33±0.23
Global South	22113	0.22±0.24	0.08±0.20	0.27±0.29
Europe	3758	0.25±0.20	0.16±0.28	0.40±0.27
Asia	8906	0.32±0.30	0.16±0.28	0.22±0.22
North America	6230	0.27±0.24	0.18±0.31	0.29±0.23
South America	7799	0.21±0.21	0.05±0.15	0.40±0.33
Oceania	524	0.22±0.27	0.24±0.35	0.47±0.35
Africa	5676	0.20±0.25	0.08±0.20	0.15±0.19
Global	32893	0.25±0.25	0.12±0.26	0.29±0.27
One-way ANOVA across infrastructure types		F(2, 32890)=3591.78, p<0.001, Cohen's f=1.00, 95% CI=[3591.13, 3592.42]		
One-way ANOVA across regions		F(5, 32887)=228.71, p<0.001, Cohen's f=0.99, 95% CI=[228.32, 229.10]	F(5, 32887)=325.55, p<0.001, Cohen's f=0.99, 95% CI=[325.08, 326.02]	F(5, 32887)=946.70, p<0.001, Cohen's f=1.00, 95% CI=[945.90, 947.50]
T-test between the Global North and Global South		t(32891)=30.86, p<0.001, Cohen's d=0.36, 95% CI=[0.08, 0.10]	t(32891)=43.14, p<0.001, Cohen's d=0.47, 95% CI=[0.12, 0.13]	t(32891)=18.33, p<0.001, Cohen's d=0.22, 95% CI=[0.05, 0.06]

Supplementary Table 2. Statistics of country-level human access to economic, social, and environmental infrastructure inequalities (measured by the Inequality index (*Inq*)) across regions. Values are presented as mean ± standard deviation. We conducted two-sided one-way ANOVA tests to examine differences in infrastructure access inequality across the three dimensions (economic, social, and environmental) and regions (Europe, Asia, North America, South America, Oceania, Africa), and a two-sided t-test to compare infrastructure access inequality between the Global North and Global South.

Region	Count	Infrastructure access inequality (*Inq*)		
		Economic	Social	Environmental
Global North	53	0.42±0.11	0.68±0.12	0.28±0.15
Global South	111	0.46±0.14	0.67±0.17	0.35±0.21
Europe	40	0.39±0.10	0.66±0.12	0.27±0.14
Asia	45	0.51±0.11	0.71±0.12	0.32±0.18
North America	15	0.44±0.15	0.67±0.16	0.49±0.14
South America	12	0.48±0.11	0.68±0.17	0.56±0.17
Oceania	4	0.38±0.22	0.55±0.27	0.62±0.22
Africa	48	0.45±0.14	0.65±0.19	0.25±0.17
Global	164	0.45±0.13	0.67±0.16	0.33±0.19
One-way ANOVA across infrastructure types		F(2, 161)=192.12, p<0.001, Cohen's f=0.99, 95% CI=[190.00, 194.25]		
One-way ANOVA across regions		F(5, 158)=5.00, p<0.001, Cohen's f=0.71, 95% CI=[4.35, 5.65]	F(5, 158)=1.15, p=0.335, Cohen's f=0.43, 95% CI=[0.84, 1.47]	F(5, 158)=14.09, p<0.001, Cohen's f=0.86, 95% CI=[12.99, 15.18]
T-test between the Global North and Global South		t(162)=-1.99, p=0.048, Cohen's d=-0.35, 95% CI=[-0.08, -0.00]	t(162)=0.68, p=0.500, Cohen's d=0.12, 95% CI=[-0.03, 0.06]	t(162)=-2.20, p=0.029, Cohen's d=-0.39, 95% CI=[-0.13, -0.02]

Supplementary Table 3. Summary of linear mixed effects regression models with disability-adjusted life years (DALYs) as the response variable. Normality was tested using the Kolmogorov-Smirnov test (D=0.135, p=0.005), indicating a significant deviation from normality. Therefore, a log transformation was applied to the response variable. Homogeneity of variances was assessed using Levene's test on residuals, and robust standard errors were applied using the sandwich estimator when p<0.05. * denotes a significance level of p<0.05. All p-values are from two-tailed tests.

	Coef.	Std.Err.	z	P>|z|	95% CI [0.025, 0.975]	
	Model I (R^2=0.45, Levene's test: F = 8.106, p = 0.005)					
ExpEco	-0.54*	0.10	-5.37	<0.001	-0.74	-0.34
ExpSoc	-0.01	0.09	-0.16	0.876	-0.18	0.15
LnPop	0.11*	0.02	6.37	<0.001	0.08	0.15
LnGDP	-0.08*	0.02	-4.11	<0.001	-0.12	-0.04
const	10.10*	0.20	51.62	<0.001	9.71	10.48
	Model II (R^2=0.34, Levene's test: F = 3.738, p = 0.055)					
GiniEco	0.69*	0.22	3.21	0.001	0.27	1.11
GiniSoc	0.18	0.20	0.91	0.362	-0.21	0.58
LnPop	0.12*	0.02	6.31	<0.001	0.08	0.15
LnGDP	-0.14*	0.02	-8.79	<0.001	-0.17	-0.11
const	10.25*	0.22	47.06	<0.001	9.82	10.68
	Model III (R^2=0.44, Levene's test: F = 6.08, p = 0.015)					
ExpEco	-0.43*	0.12	-3.59	<0.001	-0.66	-0.20
ExpSoc	-0.03	0.09	-0.32	0.748	-0.20	0.14
GiniEco	0.45*	0.22	2.03	0.043	0.01	0.88
GiniSoc	-0.07	0.21	-0.35	0.727	-0.49	0.34
LnPop	0.11*	0.02	6.07	<0.001	0.07	0.14
LnGDP	-0.09*	0.02	-4.38	<0.001	-0.13	-0.05
const	10.09*	0.20	49.54	<0.001	9.69	10.49

Supplementary Table 4. Summary of data used in this study.

Data	Format	Spatial resolution/Scale	Year	Source
Critical infrastructure data	Raster	0.1°	2020	Nirandjan, Koks[26]
Greenspace	Raster	10 m	2020	WorldCover[27]
Air pollution	Raster	0.25°	2020	GEOS-CF[29]
Heat duration	Raster	0.1°	2020	ERA5[31]
Nighttime lights	Raster	500 m	2020	VIIRS[32]
Population	Raster	100 m	2020	WorldPop
Global urban areas	Vector	>100 km^2	2020	GUB[35]
Global administrative unit layers	Vector	Country and county	/	FAO[37]
Global North and Global South country list	Tabular	Country	/	OWSD
Gross domestic product (GDP)	Tabular	Country	2020	World Bank
Human Development Index (HDI)	Tabular	Country	2020	United Nations
Health-adjusted life expectancy (HALE)	Tabular	Country	2020	IHME
Disability-adjusted life years (DALYs)	Tabular	Country	2020	IHME

Supplementary Table 5. Classification and quantile distribution of the global critical infrastructure data.

Category	CI system	Infrastructure type	Quantiles						
			50%	60%	70%	80%	90%	95%	100%
Economic	Energy	Cable	0	0	0	0	0	0	269
Economic	Energy	Line	0	0	0	0	10	18	260
Economic	Energy	Minor Line	0	0	0	0	0	0	204
Economic	Energy	Plant	0	0	0	0	0	0	42
Economic	Energy	Power Pole	0	0	0	0	0	0	17828
Economic	Energy	Power Tower	0	0	0	0	24	53	1157
Economic	Energy	Substation	0	0	0	0	0	0	1
Economic	Transportation	Airports	0	0	0	0	0	0	79
Economic	Transportation	Primary	0	0	0	0	11	18	529
Economic	Transportation	Railway	0	0	0	0	0	9	805
Economic	Transportation	Secondary	0	0	0	0	9	15	361
Economic	Transportation	Tertiary	0	4	14	31	74	130	2901
Economic	Telecommunication	Communication Tower	0	0	0	0	0	0	401
Economic	Telecommunication	Mast	0	0	0	0	0	0	131
Social	Health	Alternative	0	0	0	0	0	0	72
Social	Health	Birthing Center	0	0	0	0	0	0	6
Social	Health	Blood Donation	0	0	0	0	0	0	5
Social	Health	Clinic	0	0	0	0	0	0	481
Social	Health	Dentist	0	0	0	0	0	0	455
Social	Health	Doctors	0	0	0	0	0	0	865
Social	Health	Hospital	0	0	0	0	0	0	585
Social	Health	Laboratory	0	0	0	0	0	0	101
Social	Health	Optometrist	0	0	0	0	0	0	15
Social	Health	Pharmacy	0	0	0	0	0	0	996
Social	Health	Physiotherapist	0	0	0	0	0	0	105
Social	Health	Rehabilitation	0	0	0	0	0	0	13
Social	Education	College	0	0	0	0	0	0	14
Social	Education	Kindergarten	0	0	0	0	0	0	1
Social	Education	Library	0	0	0	0	0	0	1
Social	Education	School	0	0	0	0	0	0	10
Social	Education	University	0	0	0	0	0	0	23
Environmental	Water	Reservoir	0	0	0	0	0	0	105
Environmental	Water	Reservoir Covered	0	0	0	0	0	0	9
Environmental	Water	Water Tower	0	0	0	0	0	0	1
Environmental	Water	Water Well	0	0	0	0	0	0	1
Environmental	Water	Water Works	0	0	0	0	0	0	4
Environmental	Waste	Landfill	0	0	0	0	0	0	90
Environmental	Waste	Wastewater Treatment Plant	0	0	0	0	0	0	13
Environmental	Waste	Waste Transfer Station	0	0	0	0	0	0	7

Supplementary Table 6. Association between energy, transportation, and health infrastructure access and human health. Linear mixed effects regression models were constructed by taking health-adjusted life expectancy (HALE) as the response variable. Normality was tested using the Kolmogorov-Smirnov test, and a log transformation was applied if $p<0.05$. Homogeneity of variances was assessed using Levene's test on residuals and robust standard errors were applied using the sandwich estimator when $p<0.05$. * denotes a significance level of $p<0.05$. All p-values are from two-tailed tests.

Response variable	HALE			
Energy	8.55* (2.33)	6.0* (1.76)		13.22* (2.85)
Transportation	13.66* (2.53)		8.56* (1.98)	10.80* (3.19)
Health		8.28* (2.37)	5.42* (2.47)	5.82* (3.03)
*Energy*Transportation*	-12.05* (3.61)			-15.21* (4.95)
*Energy*Health*		-6.09 (3.57)		-8.15 (5.11)
*Transportation*Health*			10.88* (3.60)	14.92* (5.13)
LnPop	-1.78* (0.27)	-2.14* (0.29)	-2.03* (0.27)	-1.78* (0.27)
LnGDP	1.51* (0.30)	2.17* (0.31)	1.96* (0.29)	1.47* (0.30)
const	61.53* (2.90)	61.86* (3.11)	62.41* (3.09)	62.81* (2.93)
R^2	0.66	0.61	0.62	0.68

Supplementary Table 7. P-values for the Pearson correlation coefficients for access to each of the seven critical infrastructure systems at the country level. Pearson correlation coefficients were computed for each pairwise comparison (see Supplementary Fig. 7). The sample size for each correlation analysis is n=166.

	Energy	Transportation	Telecommunication	Health	Education	Water	Waste
Energy	<0.001	<0.001	<0.001	<0.001	<0.001	0.007	<0.001
Transportation	<0.001	<0.001	<0.001	<0.001	<0.001	0.117	<0.001
Telecommunication	<0.001	<0.001	<0.001	<0.001	<0.001	0.329	<0.001
Health	<0.001	<0.001	<0.001	<0.001	<0.001	0.240	<0.001
Education	<0.001	<0.001	<0.001	<0.001	<0.001	0.020	<0.001
Water	0.007	0.117	0.329	0.240	0.020	<0.001	0.006
Waste	<0.001	<0.001	<0.001	<0.001	<0.001	0.006	<0.001

Supplementary references

1. Smith A. *The Wealth of Nations*. W. Strahan and T. Cadell (1776).
2. Jerome A. *Infrastructure in Africa: the record*. African Development Bank (1999).
3. Hirschman AO. *The Strategy of Economic Development*. Yale University Press (1958).
4. Buhr W. What is infrastructure? (2003).
5. Fulmer J. What in the World is Infrastructure? *PEI Infrastructure Investor*, 30-32 (2009).
6. Baskakova IV, Malafeev N. The Concept of Infrastructure: Definition, Classification and Methodology for Empirical Evaluation.) (2017).
7. Gallais C, Filiol E. Critical Infrastructure: Where Do We Stand Today? A Comprehensive and Comparative Study of the Definitions of a Critical Infrastructure. *Journal of Information Warfare* **16**, 64-87 (2017).
8. Torrisi G. Public infrastructure: definition, classification and measurement issues. (2009).
9. Biehl D. The Role of Infrastructure in Regional Development. In: *Infrastructure and Regional Development* (ed Vickerman RW). Pion (1991).
10. Di Palma M, Mazziotta C. *Infrastrutture e sviluppo. Primi risultati: indicatori quantitativi a confronto (1987–95)*. Quaderni sul Mezzogiorno e le politiche territoriali (1998).
11. Hansen NM. The Structure and Determinants of Local Public Investment Expenditures. *The Review of Economics and Statistics* **47**, 150-162 (1965).
12. Fourie J. ECONOMIC INFRASTRUCTURE: A REVIEW OF DEFINITIONS, THEORY AND EMPIRICS. *South African Journal of Economics* **74**, 530-556 (2006).
13. Davern M*, et al.* Using spatial measures to test a conceptual model of social infrastructure that supports health and wellbeing. *Cities & Health* **1**, 194-209 (2017).
14. Rogelj V, Bogataj D. Social infrastructure of Silver Economy: Literature review and Research agenda. *IFAC-PapersOnLine* **52**, 2680-2685 (2019).
15. Challoumis C. The Role of Infrastructure in Economic Development.).
16. Sturm JE, Jacobs J. Productivity Impacts of Infrastructure Investment in the Netherlands 1853–1913. (1995).
17. Jochimsen R. *Theorie der Infrastruktur: Grundlagen der marktwirtschaftlichen Entwicklung*. J.C.B. Mohr (1966).
18. Vaughan-Morris G. Strategic Infrastructure: Steps to Prioritize and Deliver Infrastructure Effectively and Efficiently. (2012).
19. Wang J, Banzhaf E. Towards a better understanding of Green Infrastructure: A critical review. *Ecological Indicators* **85**, 758-772 (2018).
20. Ying J, Zhang X, Zhang Y, Bilan S. Green infrastructure: systematic literature review. *Economic Research-Ekonomska Istraživanja* **35**, 343-366 (2022).
21. Makropoulos CK, Butler D. Distributed Water Infrastructure for Sustainable Communities. *Water Resources Management* **24**, 2795-2816 (2010).
22. Meeks RC. Water Works: The Economic Impact of Water Infrastructure. *Journal of Human Resources* **52**, 1119 (2017).
23. Frischmann BM. Environmental Infrastructure. *Ecology Law Quarterly* **35**, 151-178 (2008).
24. Browder G, Ozment S, Rehberger Bescos I, Gartner T, Lange G-M. Integrating Green and Gray: Creating Next Generation Infrastructure. (2019).
25. OECD, Bank TW, Programme UNE. *Financing Climate Futures: Rethinking Infrastructure* (2018).

26. Nirandjan S, Koks EE, Ward PJ, Aerts JC. A spatially-explicit harmonized global dataset of critical infrastructure. *Scientific Data* **9**, 150 (2022).
27. Zanaga D*, et al.* ESA WorldCover 10 m 2021 v200. (2022).
28. Chen B, Wu S, Song Y, Webster C, Xu B, Gong P. Contrasting inequality in human exposure to greenspace between cities of Global North and Global South. *Nature Communications* **13**, 4636 (2022).
29. Keller CA*, et al.* Description of the NASA GEOS Composition Forecast Modeling System GEOS-CF v1.0. *Journal of Advances in Modeling Earth Systems* **13**, e2020MS002413 (2021).
30. Gorelick N, Hancher M, Dixon M, Ilyushchenko S, Thau D, Moore R. Google Earth Engine: Planetary-scale geospatial analysis for everyone. *Remote Sensing of Environment* **202**, 18-27 (2017).
31. Hersbach H*, et al.* The ERA5 global reanalysis. *Quarterly Journal of the Royal Meteorological Society* **146**, 1999-2049 (2020).
32. Elvidge CD, Baugh K, Zhizhin M, Hsu FC, Ghosh T. VIIRS night-time lights. *International Journal of Remote Sensing* **38**, 5860-5879 (2017).
33. Elvidge CD, Baugh KE, Zhizhin M, Hsu F-C. Why VIIRS data are superior to DMSP for mapping nighttime lights. *Proceedings of the Asia-Pacific Advanced Network* **35**, 62 (2013).
34. Elvidge CD, Zhizhin M, Ghosh T, Hsu F-C, Taneja J. Annual time series of global VIIRS nighttime lights derived from monthly averages: 2012 to 2019. *Remote Sensing* **13**, 922 (2021).
35. Li X*, et al.* Mapping global urban boundaries from the global artificial impervious area (GAIA) data. *Environmental Research Letters* **15**, 094044 (2020).
36. Tu Y, Chen B, Yang J, Xu B. Olympic effects on reshaping urban greenspace of host cities. *Landscape and Urban Planning* **230**, 104615 (2023).
37. Food and Agriculture Organization of the United Nations. Global Administrative Unit Layers (GAUL).) (2015).
38. Gonzalez CG. Environmental justice, human rights, and the global south. *Santa Clara Journal of International Law* **13**, 151 (2015).
39. Stevens FR, Gaughan AE, Linard C, Tatem AJ. Disaggregating census data for population mapping using random forests with remotely-sensed and ancillary data. *PLOS One* **10**, e0107042 (2015).
40. Doxsey-Whitfield E*, et al.* Taking advantage of the improved availability of census data: a first look at the gridded population of the world, version 4. *Papers in Applied Geography* **1**, 226-234 (2015).
41. Dobson JE, Bright EA, Coleman PR, Durfee RC, Worley BA. LandScan: a global population database for estimating populations at risk. *Photogrammetric Engineering and Remote Sensing* **66**, 849-857 (2000).
42. World Health Organization. World health statistics 2020. (2020).
43. Murray CJ, Lopez AD. *The global burden of disease: a comprehensive assessment of mortality and disability from diseases, injuries, and risk factors in 1990 and projected to 2020: summary*. World Health Organization (1996).
44. Logan TM, Anderson MJ, Williams TG, Conrow L. Measuring inequalities in urban systems: An approach for evaluating the distribution of amenities and burdens. *Computers, Environment and Urban Systems* **86**, 101590 (2021).
45. Gastwirth JL. The estimation of the Lorenz curve and Gini index. *The review of economics and statistics*, 306-316 (1972).
46. Lambert PJ, Aronson JR. Inequality decomposition analysis and the Gini coefficient revisited. *The Economic Journal* **103**, 1221-1227 (1993).

47. Brelsford C, Lobo J, Hand J, Bettencourt LMA. Heterogeneity and scale of sustainable development in cities. *Proceedings of the National Academy of Sciences* **114**, 8963-8968 (2017).
48. Pandey B, Brelsford C, Seto KC. Infrastructure inequality is a characteristic of urbanization. *Proceedings of the National Academy of Sciences* **119**, e2119890119 (2022).
49. Zhou Y*, et al.* Satellite mapping of urban built-up heights reveals extreme infrastructure gaps and inequalities in the Global South. *Proceedings of the National Academy of Sciences* **119**, e2214813119 (2022).
