# Supplementary material for: Inequality in infrastructure access and its association with health disparities
Source: Nat Hum Behav. 2025 May 22;9(8):1669–82. doi: 10.1038/s41562-025-02208-3 (PMC12367552; doi:10.1038/s41562-025-02208-3)
Supplement: Supplementary file 2 — Reporting Summary [file 41562_2025_2208_MOESM2_ESM.pdf]

nature portfolio

Corresponding author(s): Bin Chen and Bing Xu

Last updated by author(s): Feb 17, 2025

Reporting Summary

Nature Portfolio wishes to improve the reproducibility of the work that we publish. This form provides structure for consistency and transparency in reporting. For further information on Nature Portfolio policies, see our Editorial Policies and the Editorial Policy Checklist.

Statistics

For all statistical analyses, confirm that the following items are present in the figure legend, table legend, main text, or Methods section.

n/a	Confirmed	
☐	☒	The exact sample size (*n*) for each experimental group/condition, given as a discrete number and unit of measurement
☐	☒	A statement on whether measurements were taken from distinct samples or whether the same sample was measured repeatedly
☐	☒	The statistical test(s) used AND whether they are one- or two-sided<br>*Only common tests should be described solely by name; describe more complex techniques in the Methods section.*
☐	☒	A description of all covariates tested
☐	☒	A description of any assumptions or corrections, such as tests of normality and adjustment for multiple comparisons
☐	☒	A full description of the statistical parameters including central tendency (e.g. means) or other basic estimates (e.g. regression coefficient) AND variation (e.g. standard deviation) or associated estimates of uncertainty (e.g. confidence intervals)
☐	☒	For null hypothesis testing, the test statistic (e.g. *F*, *t*, *r*) with confidence intervals, effect sizes, degrees of freedom and *P* value noted<br>*Give P values as exact values whenever suitable.*
☒	☐	For Bayesian analysis, information on the choice of priors and Markov chain Monte Carlo settings
☒	☐	For hierarchical and complex designs, identification of the appropriate level for tests and full reporting of outcomes
☐	☒	Estimates of effect sizes (e.g. Cohen's *d*, Pearson's *r*), indicating how they were calculated

Our web collection on statistics for biologists contains articles on many of the points above.

Software and code

Policy information about availability of computer code

Data collection	No software was used for data collection.
Data analysis	All data processing and analysis were conducted using Google Earth Engine, Python (version 3.11), and ArcMap (version 10.8).

For manuscripts utilizing custom algorithms or software that are central to the research but not yet described in published literature, software must be made available to editors and reviewers. We strongly encourage code deposition in a community repository (e.g. GitHub). See the Nature Portfolio guidelines for submitting code & software for further information.

Data

Policy information about availability of data

All manuscripts must include a data availability statement. This statement should provide the following information, where applicable:

- Accession codes, unique identifiers, or web links for publicly available datasets
- A description of any restrictions on data availability
- For clinical datasets or third party data, please ensure that the statement adheres to our policy

Data Availability
A number of public datasets used in this study can be accessed via the Google Earth Engine platform (https://earthengine.google.com), specifically including:
• The Harmonized Global Critical Infrastructure dataset (https://gee-community-catalog.org/projects/cisi/)
• The WorldCover Global Land Cover map for 2020 (https://developers.google.com/earth-engine/datasets/catalog/ESA_WorldCover_v100)

• NASA’s Goddard Earth Observing System Composition Forecast (GEOS-CF) data (https://developers.google.com/earth-engine/datasets/catalog/NASA_GEOS-CF_v1_rpl_tavg1hr)
• The ERA5-Land Daily Aggregated Climate Reanalysis data (https://developers.google.com/earth-engine/datasets/catalog/ECMWF_ERA5_LAND_DAILY_AGGR)
• The WorldPop Global Project Population Data (https://developers.google.com/earth-engine/datasets/catalog/WorldPop_GP_100m_pop)
The annual global VIIRS nighttime lights (VNL) V2 product is from the Earth Observation Group (https://eogdata.mines.edu/products/vnl/#annual_v2). The global urban boundary (GUB) datasets are from Pengcheng Laboratory (https://data-starcloud.pcl.ac.cn/). The Global Administrative Unit Layers (GAULs) are from the Food and Agriculture Organization of the United Nations (https://data.apps.fao.org/). The list of Global South countries was obtained from the Organization for Women in Science for the Developing World (https://owsd.net/). Data on gross domestic product (GDP) are from the World Bank (https://data.worldbank.org). Data on Human Development Index (HDI) are from the United Nations Development Programme (https://hdr.undp.org/). Health data on health-adjusted life expectancy (HALE) and disability-adjusted life years (DALYs) are from the Institute for Health Metrics and Evaluation at the University of Washington (https://vizhub.healthdata.org/gbd-results/).
The resulting maps of economic, social, and environmental infrastructure, along with data on infrastructure access, infrastructure access inequality, and health outcomes, have been deposited in an open repository at https://figshare.com/projects/Infrastructure_inequality/237854.

Research involving human participants, their data, or biological material

Policy information about studies with human participants or human data. See also policy information about sex, gender (identity/presentation), and sexual orientation and race, ethnicity and racism.

Reporting on sex and gender	n/a
Reporting on race, ethnicity, or other socially relevant groupings	n/a
Population characteristics	n/a
Recruitment	n/a
Ethics oversight	n/a

Note that full information on the approval of the study protocol must also be provided in the manuscript.

Field-specific reporting

Please select the one below that is the best fit for your research. If you are not sure, read the appropriate sections before making your selection.

☐ Life sciences ☒ Behavioural & social sciences ☐ Ecological, evolutionary & environmental sciences

For a reference copy of the document with all sections, see nature.com/documents/nr-reporting-summary-flat.pdf

Behavioural & social sciences study design

All studies must disclose on these points even when the disclosure is negative.

Study description	We combined existing critical infrastructure datasets, land cover products, air pollution data, and reanalysis climate data to generate global maps of economic, social, and environmental infrastructure distributions. We employed a population-weighted exposure model to reveal spatial differences in human access to infrastructure and the associated inequality levels between Global North and Global South countries. Additionally, we performed mixed-effects regression analysis to explore the relationship between infrastructure access/inequality and human health.
Research sample	We analyzed infrastructure access inequality and the associated health disparities across 166 countries worldwide, including 54 in the Global North and 112 in the Global South. This study sample is representative, covering more than 99% of the global population (~7.77 billion). This broad geographic coverage enables a comprehensive analysis of infrastructure access inequality and associated health disparities across diverse economic and regional contexts.
Sampling strategy	We assessed human access to infrastructure at both the country and county levels. Infrastructure access inequalities and regression analyses between infrastructure access/inequality and human health were conducted at the country level. The sample size was not predetermined using a formal statistical method. Instead, only countries with both infrastructure and population distribution were considered, resulting in a final sample of 166 countries. This sample size, representing over 99% of the global population, is sufficient to capture patterns of human access and inequality.
Data collection	All the data used in this study were from international organizations, open-source databases and peer-reviewed papers. These data were accessed through direct website downloads or the Google Earth Engine platform (https://earthengine.google.com).
Timing	We used the year 2020 as a baseline to elucidate the results and findings. Data were collected between March 2023 and May 2023.
Data exclusions	n/a
Non-participation	No participants were involved in the study.

Randomization	Randomization was not relevant to this study because our analysis was based on multi-source geospatial datasets rather than a subset of sampled observations. We included all countries with available infrastructure and population distribution data, ensuring broad coverage rather than selecting a random sample. This approach allows for a systematic and representative assessment of infrastructure access and inequality across diverse economic and regional contexts.

Reporting for specific materials, systems and methods

We require information from authors about some types of materials, experimental systems and methods used in many studies. Here, indicate whether each material, system or method listed is relevant to your study. If you are not sure if a list item applies to your research, read the appropriate section before selecting a response.

Materials & experimental systems

n/a	Involved in the study	
☒	☐	Antibodies
☒	☐	Eukaryotic cell lines
☒	☐	Palaeontology and archaeology
☒	☐	Animals and other organisms
☒	☐	Clinical data
☒	☐	Dual use research of concern
☒	☐	Plants

Methods

n/a	Involved in the study	
☒	☐	ChIP-seq
☒	☐	Flow cytometry
☒	☐	MRI-based neuroimaging

Plants

Seed stocks	*Report on the source of all seed stocks or other plant material used. If applicable, state the seed stock centre and catalogue number. If plant specimens were collected from the field, describe the collection location, date and sampling procedures.*
Novel plant genotypes	*Describe the methods by which all novel plant genotypes were produced. This includes those generated by transgenic approaches, gene editing, chemical/radiation-based mutagenesis and hybridization. For transgenic lines, describe the transformation method, the number of independent lines analyzed and the generation upon which experiments were performed. For gene-edited lines, describe the editor used, the endogenous sequence targeted for editing, the targeting guide RNA sequence (if applicable) and how the editor was applied.*
Authentication	*Describe any authentication procedures for each seed stock used or novel genotype generated. Describe any experiments used to assess the effect of a mutation and, where applicable, how potential secondary effects (e.g. second site T-DNA insertions, mosiacism, off-target gene editing) were examined.*
